# Estimation of Minimal Clinically Important Difference for Tinnitus Handicap Inventory and Tinnitus Functional Index

**DOI:** 10.1002/ohn.1217

**Published:** 2025-03-20

**Authors:** Milena Engelke, Laura Basso, Berthold Langguth, Florian Zeman, Winfried Schlee, Stefan Schoisswohl, Rilana Cima, Dimitris Kikidis, Jose Antonio Lopez‐Escamez, Petra Brüggemann, Birgit Mazurek, Jorge Piano Simões

**Affiliations:** ^1^ Department of Psychiatry and Psychotherapy University of Regensburg Regensburg Germany; ^2^ Center for Clinical Studies, University Hospital Regensburg Regensburg Germany; ^3^ Institute for Information and Process Management Eastern Switzerland University of Applied Sciences St. Gallen Switzerland; ^4^ Department of Human Sciences Institute of Psychology Universitaet der Bundeswehr München Neubiberg Germany; ^5^ Department of Health Psychology Katholieke Universiteit Leuven Leuven Belgium; ^6^ First Department of Otorhinolaryngology–Head and Neck Surgery Hippocrateion General Hospital National and Kapodistrian University of Athens Athens Greece; ^7^ Division of Otolaryngology, Department of Surgery Instituto de Investigacion Biosanitaria, ibs.GRANADA Universidad de Granada Granada Spain; ^8^ Meniere's Disease Neuroscience Research Program, Faculty of Medicine and Health School of Medical Sciences, The Kolling Institute University of Sydney Sydney New South Wales Australia; ^9^ Tinnitus Center, Charité – Universitätsmedizin Berlin (Corporate Member of Freie Universität Berlin and Humboldt‐Universität zu Berlin) Berlin Germany; ^10^ Department of Psychology, Health and Technology University of Twente Enschede The Netherlands

**Keywords:** minimal clinically important difference, tinnitus, Tinnitus Functional Index, Tinnitus Handicap Inventory

## Abstract

**Objective:**

The minimal clinically important difference (MCID) represents the smallest change in treatment outcome deemed clinically meaningful. This study estimates the MCID for 2 widely used tinnitus measures: the Tinnitus Handicap Inventory (THI) and the Tinnitus Functional Index (TFI), using anchor‐based approaches while accounting for baseline severity and time interval.

**Study Design:**

A multi‐center randomized clinical trial.

**Setting:**

European tinnitus centers.

**Methods:**

Anchor‐based approaches, including the effect size, receiver‐operating characteristics, and ΔTHI/TFI methods, were employed to determine the MCID. The “minimally improved” category of the Clinical Global Impression Scale‐Improvement (CGI‐I) served as the anchor. The standard error of measurement was used to assess random variation.

**Results:**

For the THI, MCID estimates ranged from 7.8 to 12, with a point estimate of 11 after 12 weeks of treatment (N = 364). For the TFI, MCID estimates ranged from 7.3 to 9.4, with a point estimate of 9 points after 12 weeks (N = 359). Both measures indicated that higher baseline severity and longer time intervals required greater score reduction for clinical relevance.

**Conclusion:**

This study highlights the context‐specific nature of MCID values for tinnitus measures and emphasizes the need for consensus on optimal anchor‐based approaches to improve comparability.

The minimal clinically important difference (MCID) is defined as the smallest change in a patient‐reported outcome measure (PROM) that is meaningful to patients.^1^ It is important because it helps clinicians and researchers to understand whether a particular intervention provides at least a minimally meaningful benefit to patients.^2^

Approaches to estimate the MCID can be either distribution‐based or anchor‐based. Each methodology has strengths and limitations.^2^, ^3^ Distribution‐based methods estimate the MCID based on the statistical properties of the sample and can be used to estimate how much change is required to go beyond a certain level of random variation. However, they do not rely on external criteria and therefore do not provide information on clinical relevance.^4^, ^5^, ^6^, ^7^ In contrast, anchor‐based methods use external criteria, such as patient‐reported ratings of improvement or clinical judgments, to determine the MCID and thus provide a more patient‐centered and clinically relevant estimate.^5^, ^6^, ^7^, ^8^

Tinnitus is often associated with sensorineural hearing loss and neuroplastic changes in the auditory pathway.^9^, ^10^ Its impact on daily life can range from mild to severe. Severe tinnitus often affects emotional well‐being and cognitive function,^11^ with treatments aimed at reducing tinnitus distress and associated symptoms. Their effect is measured in clinical trials using PROMs, which estimate the impact of tinnitus on daily living.^12^ Both the Tinnitus Handicap Inventory (THI) and the Tinnitus Functional Index (TFI) belong to the most widespread questionnaires in clinical research and practice.^12^, ^13^, ^14^

Upon development, a change of 20 points in the THI was defined as minimal detectable change based on test–retest reliability.^15^ An estimation of the MCID for the THI involving anchor‐based methods has been conducted later on.^16^ Data from 210 tinnitus patients undergoing various therapies yielded an MCID estimate of 6 to 16 (point estimate: 7) for THI score reduction, which has been frequently cited in clinical trials but lacks replication.^4^, ^16^, ^17^, ^18^, ^19^

For the TFI, an MCID of 13 points was originally proposed,^20^ but has since been estimated several times with results ranging from 4.8 to 22.4 points (Table 1). Explanations for this large range include heterogeneous methodology as well as variability in chosen anchors and time frames.^20^, ^21^, ^22^, ^23^, ^24^ Clinical trials, which determined responder rates using an MCID of 7 for the THI^16^ and 13 for the TFI,^20^ revealed highly discrepant results for the 2 questionnaires.^18^, ^25^

**Table 1:** Overview of Minimal Clinically Important Difference (MCID) Estimates for TFI in the Literature

Reference	MCID	Method	Type	Intervention	Timeframe	N	Mean TFI baseline
Meikle et al (2012)^20^	13 points	Difference in TFI score reduction between the “much to moderately improved” group and the “unchanged” group = 14	Anchor‐based	Different interventions	3 mo	150	*M* = 54.4 (SD = 24.7; N = 347)
		One‐half SD of TFI baseline scores = 12.35	Distribution‐ based			347	
Fackrell et al (2016)^21^	22.4 points	Smallest detectable change (SDC)	Distribution‐ based	Sound therapy	15 d	44	*M* = 45.3 (SD = 20.1; N = 44)
Chandra et al (2018)^22^	4.8 points	Smallest detectable change (SDC)	Distribution‐ based	No intervention	2 wk	40	*M* = 47.3 (SD = 17.0; N = 40)
Skarżyński et al (2018)^23^	8.8 points	Mean TFI change of the “minimally improved” group = 8.7	Anchor‐based	Stapedotomy^a^	3 mo	95	*M* = 31.83 (SD = 20.79; N = 95)
		ROC discrimination between “much/very much improvement” versus “no change” (criterion: minimum of the sum of 1‐sensitivity and 1‐ specificity) = 8.8					
Fackrell et al (2022)^24^	14 points	Difference in TFI score reduction between the “moderately improved” group and “no change” group = 12.6 (3 mo); 14.6 (6 mo); 13.7 (9 mo)	Anchor‐based	Different interventions	3, 6, and 9 mo	196/175/165	*M* = 50.8 (SD = 25.1; N = 50)
		Smallest detectable change (SDC) = 14.2	Distribution‐ based			50	

The N column refers to the number of participants included in the respective MCID analysis (not necessarily equal to the study sample). For the mean TFI baseline scores, the number of participants is given in brackets.

Abbreviations: ROC, receiver‐operating characteristics; SD, standard deviation; TFI, Tinnitus Functional Index.

^a^ Stapedotomy is a surgical procedure modifying/replacing the stapes bone in the middle ear to improve hearing.

The present study aims to gain new insights into what constitutes an appropriate MCID measure for both THI and TFI, using a multicenter randomized clinical trial (RCT) sample of tinnitus patients^26^ and conceptually selected anchor‐based MCID measures, and to compare our findings with previous studies. The methodology includes different anchor‐based methods to estimate the MCID, while the standard error of measurement (SEM) is used to compare the anchor‐based estimates against random variation. To account for heterogeneous findings, the influence of time interval and baseline severity was further investigated as the MCID is known to be highly context‐specific.^4^, ^5^, ^24^, ^27^

## Methods

### Data

Data from a multi‐center RCT were used that investigated the effect of single and combination therapies on tinnitus (UNITI project).^26^, ^28^, ^29^ Ethical approval was obtained at each of the 5 clinical sites (names are given on page 1 of the Supplemental Information, available online) and patients provided written informed consent before trial participation.^26^ Patients received Cognitive Behavioral Therapy, Hearing Aids, Structured Counseling, or Sound Therapy either as a single treatment or as a combination of 2 of these treatments for 12 weeks. Measurements were taken before treatment, after 6, 12, and 36 weeks. The main results of the RCT with 461 patients is reported elsewhere.^28^ The present analysis is based on complete cases; thus, only patients who fully completed the outcome of interest (THI or TFI) at the respective time points, as well as the anchor (Clinical Global Impression Scale‐Improvement [CGI‐I]), were included.

### Participants

Patients were included if their main complaint was tinnitus, had chronic tinnitus (≥6 months), at least mild tinnitus handicap (THI ≥ 18), and had not started any other tinnitus‐related treatments in the last 3 months before the study started. Details on eligibility and other criteria can be found in the study protocol.^26^

### Measures

The THI is frequently used in clinical research and practice to quantify tinnitus‐related handicaps.^14^, ^30^ It consists of 25 items and a functional, emotional, and catastrophic subscale. Total scores range from 0 to 100. Validation indicated high internal consistency and high convergent validity with the Tinnitus Questionnaire.^30^, ^31^, ^32^ Although originally not designed as outcome measure, the THI is sensitive to treatment‐related changes.^25^, ^33^

The TFI measures the functional impact of tinnitus and consists of 25 items.^20^ It includes 8 subscales: cognitive interference, auditory perceptual difficulties, intrusiveness, sleep disturbances, disturbances of relaxation, quality of life impairment, emotional distress, and reduced sense of control. The total score ranges from 0 and 100. The TFI shows high internal consistency and high construct validity with the THI.^34^

The CGI‐I^35^ modified for tinnitus was chosen as anchor criterion. Within 1 question, patients are asked to rate the overall improvement of their tinnitus complaints compared to before the treatment has started on a 7‐point Likert scale (1—“very much better,” 2—“much better,” 3—“minimally better,” 4—“no change,” 5—“minimally worse,” 6—“much worse,” 7—“very much worse”). The CGI‐I has been used as an anchor to determine the MCID in the tinnitus field.^16^, ^24^, ^27^ We used the category “minimally better” (CGI‐I = 3) compared to the “no change” group (CGI‐I = 4) as the MCID anchor.^4^

### Statistical Analysis

THI/TFI baseline and change values (ΔTHI/TFI = baseline score – 6/12/36 weeks score) were stratified according to CGI‐I scores. Spearman correlation coefficients for CGI‐I and ΔTHI/TFI were computed. The SEM (SEM = $\mathrm{SD}\sqrt{1-R}$) was calculated with the standard deviation (SD) of the baseline score and *R* being the test–retest reliability of the respective scale (*R*_THI_ = 0.93^30^; *R*_TFI_ = 0.89; see page 1 of the Supplemental Information and Supplemental Table S1, available online).

The main analysis focused on the change from baseline to week 12. Secondary analyses included change from baseline to weeks 6 and 36, as well as the change from baseline to week 12 for different baseline levels, that is, mild (or very mild) tinnitus impact (THI ≤ 36; TFI ≤ 31), moderate tinnitus impact (38 ≤ THI ≤ 56; 32 ≤ TFI ≤ 53), and severe (or catastrophic) tinnitus impact (THI ≥ 58; TFI ≥ 54). All analyses were performed in R (version 4.2.2). The following methods were used to determine the MCID.

#### Effect Size Method

**Figure 1:**
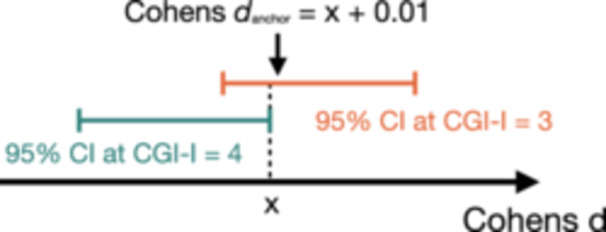
Graphical illustration of Cohen's *d*_anchor._ The first effect size value at the lower CI end of the anchor group outside the upper CI limit of the comparison group was chosen as the MCID. CGI‐I, Clinical Global Impression Scale‐Improvement; CI, confidence interval; MCID, minimal clinically important difference.

Cohen's *d* effect sizes and their corresponding 95% confidence intervals (CIs; noncentrality parameter method) of score change were calculated for the corresponding CGI‐I categories (R package *effectsize*).^36^ The first effect size (on the second decimal) at the lower CI end of the anchor category (“minimally better”) being outside the CI of the comparison category (“no change”) was chosen (Cohen's *d*_anchor_; Figure 1). This effect size was multiplied by the SD of the ΔTHI/TFI to obtain the MCID estimate. We chose this method because of its precise approach to separating the anchor group from the comparison group based on their respective CIs. It was also used by Zeman et al.^16^

$$\text{Cohens }d=\frac{{\mathrm{Mean}}_{\mathrm{baseline}}-{\mathrm{Mean}}_{\mathrm{final}\;\mathrm{visit}}}{({\mathrm{SD}}_{\mathrm{baseline}}+{\mathrm{SD}}_{\mathrm{final}\;\mathrm{visit}})/2}$$

$${\mathrm{MCID}}_{\mathrm{Effect}\;\mathrm{size}}={\mathrm{Cohens}\;d}_{\mathrm{anchor}}*{\mathrm{SD}}_{\mathrm{\Delta THI}/\mathrm{TFI}}$$

#### ROC Method

A receiver‐operating characteristics (ROC) curve was used to discriminate the “minimally better” group from the “no change” group according to the ΔTHI/TFI (R package *ROCit*).^37^ The optimal threshold (ie, MCID) was determined by equally considering sensitivity and specificity based on the Euclidian method.^38^

$${\mathrm{MCID}}_{\mathrm{ROC}}=\min\left\{{\left(1-\mathrm{sensitivity}\right)}^{2}+{\left(1-\mathrm{specificity}\right)}^{2}\right\}$$

#### ΔTHI/TFI Method

The MCID was determined by subtracting the mean ΔTHI/TFI of the “no change” group from the mean ΔTHI/TFI of the “minimally better” group.^3^

$${\mathrm{MCID}}_{\mathrm{\Delta THI}/\mathrm{TFI}}={\mathrm{Mean}}_{\mathrm{\Delta minimallybetter}}-{\mathrm{Mean}}_{\mathrm{\Delta nochange}}$$

## Results

### Participants

Complete data at baseline and after 12 weeks were available for 364 patients for the THI and 359 patients for the TFI (Table 2).

**Table 2:** Patient Characteristics at Baseline

	N (%)/mean ± SD	
Characteristic	THI sample (N = 364)	TFI sample (N = 359)
Sex, no. (%)		
Female	156 (42.9%)	154 (42.9%)
Male	208 (57.1%)	205 (57.1%)
Age (y)	51.2 ± 12.5	51.2 ± 12.5
Tinnitus duration (mo)*	96.0 ± 156*	96.0 ± 156*
THI/TFI score	48.3 ± 19.7	49.1 ± 20.7
THI/TFI severity, no. (%)		
Very mild handicap/not a problem	4 (1.1%)	25 (7.0%)
Mild handicap/small problem	117 (32.1%)	56 (15.6%)
Moderate handicap/moderate problem	129 (35.4%)	122 (34.0%)
Severe handicap/big problem	72 (19.8%)	102 (28.4%)
Catastrophic handicap/very big problem	42 (11.5%)	54 (15.0%)
PHQ‐9 score	7.3 ± 5.0	7.3 ± 5.0

Only * for tinnitus duration median ± IQR is used due to non‐normally distributed data (all other values are means ± SD). THI severity: very mild handicap = 0 to 16; mild handicap = 18 to 36; moderate handicap = 38 to 56; severe handicap = 58 to 76; catastrophic handicap = 78 to 100.^39^ TFI severity: not a problem = 0 to 17; small problem = 18 to 31; moderate problem = 32 to 53; big problem = 54 to 72; very big problem = 73 to 100.^20^ Inclusion criteria were verified at a screening visit; baseline values may deviate.

Abbreviations: PHQ‐9, Patient Health Questionnaire for Depression; SD, standard deviation; TFI, Tinnitus Functional Index; THI, Tinnitus Handicap Inventory.

### Descriptive Statistics

**Figure 2:**
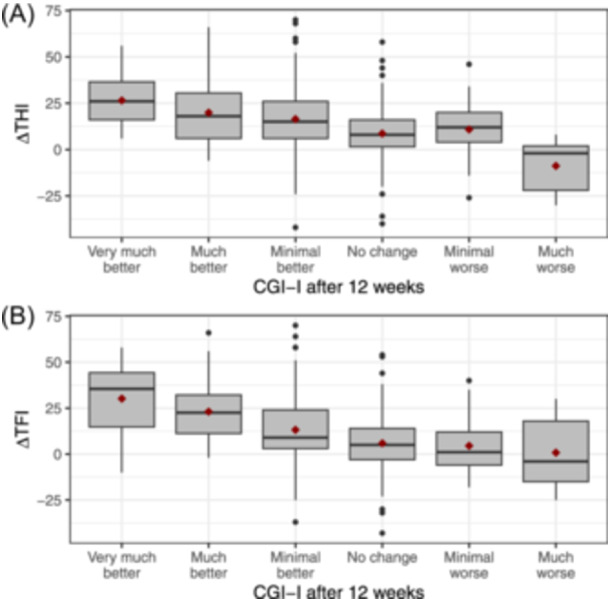
ΔTHI/TFI distribution at CGI‐I after 12 weeks. (A) ΔTHI distribution. (B) ΔTFI distribution. Error bars indicate whiskers (standard boxplot), and red squares show the group mean. CGI‐I, Clinical Global Impression Scale‐Improvement; TFI, Tinnitus Functional Index; THI, Tinnitus Handicap Inventory.

CGI‐I and ΔTHI/TFI distributions are reported in 3, 4 and 4 and Figure 2. Patients reported that their tinnitus complaints had “not changed” had a mean improvement of 8.7 (SD = 14.5) in THI score and 5.9 points (SD = 15.8) in TFI score. Patients reported that their tinnitus complaints were “minimally better” and had a mean improvement of 16.4 (SD = 18) in THI score and 13.2 points (SD = 17.4) in TFI score. The correlation with the CGI‐I is weak for the ΔTHI (*ρ* = −0.29, *P* < .001) and moderate for the ΔTFI (*ρ* = −0.40, *P* < .001).

**Table 3:** CGI‐I Groups, Baseline THI, and ΔTHI After 12 Weeks

CGI‐I groups	N (%)	Baseline THI (mean ± SD)	ΔTHI (mean ± SD)
Very much better	16 (4.4%)	49.8 ± 14.5	26.5 ± 14.6
Much better	56 (15.4%)	49.4 ± 20	19.9 ± 17.3
Minimally better	112 (30.8%)	51.2 ± 20.3	16.4 ± 18.0
No change	132 (36.3%)	45.4 ± 20.8	8.7 ± 14.5
Minimally worse	43 (11.8%)	48.4 ± 15.9	10.8 ± 13.2
Much worse	5 (1.4%)	38 ± 8.6	‐8.8 ± 16.3
Very much worse	0	‐	‐

Plus–minus values are means ± SD. ΔTHI = baseline THI – THI after 12 weeks.

Abbreviations: CGI‐I, Clinical Global Impression Scale‐Improvement; SD, standard deviation; THI, Tinnitus Handicap Inventory.

**Table 4:** CGI‐I Groups, Baseline TFI, and ΔTFI After 12 Weeks

CGI‐I groups	N (%)	Baseline TFI (mean ± SD)	ΔTFI (mean ± SD)
Very much better	16 (4.5%)	49.8 ± 18.5	30.2 ± 19.4
Much better	56 (15.6%)	53.6 ± 18.5	23.1 ± 16.1
Minimally better	112 (31.2%)	49.3 ± 21	13.2 ± 17.4
No change	129 (35.9%)	47 ± 21.9	5.9 ± 15.8
Minimally worse	41 (11.4%)	47.9 ± 21	4.5 ± 14.1
Much worse	5 (1.4%)	51.8 ± 9	0.8 ± 22.8
Very much worse	0	‐	‐

Plus–minus values are means ± SD. ΔTFI = baseline TFI – TFI after 12 weeks.

Abbreviations: CGI‐I, Clinical Global Impression Scale‐Improvement; SD, standard deviation; TFI, Tinnitus Functional Index.

### MCID Estimation

**Figure 3:**
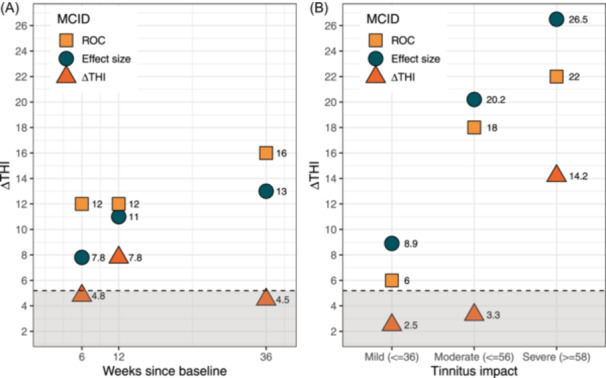
THI‐MCID. (A) MCID results by time interval. (B) MCID results by tinnitus severity at baseline. The dashed line represents the standard error of measurement (results below cannot be interpreted). MCID, minimal clinically important difference; THI, Tinnitus Handicap Inventory.

**Figure 4:**
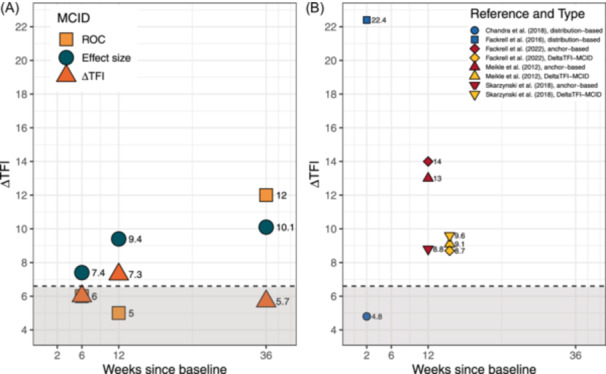
TFI‐MCID by time interval. (A) MCID results by time interval. (B) Literature MCID results. ΔTFI‐MCID calculation for better comparability: difference between no change group and the minimally better group. The dashed line represents the standard error of measurement. MCID, minimal clinically important difference; TFI, Tinnitus Functional Index.

The MCID results for the different anchor‐based methods are shown in Figures 3A and 4A. The SEM was 5.2 for the THI and 6.9 for the TFI; values below this threshold cannot be interpreted.

For the THI, effect size estimation of the “minimally better” CGI‐I group yielded a large effect (Cohen's *d* = 0.82, 95% CI: 0.54, 1.09), which was compared with the “no change” group (Cohen's *d* = 0.40, 95% CI: 0.16, 0.64). Cohen's *d*_anchor_ = 0.65 was multiplied by the pooled SD of the ΔTHI (16.9), which resulted in an effect size MCID of 11.0. The ROC analysis yielded an area under the curve (AUC) value of 0.64 and an optimal cut‐off threshold of 12, which represents the ROC‐MCID. To determine the ΔTHI‐MCID, the mean change score of the “minimally better” CGI‐I group (*M* = 16.43) was subtracted by the mean change score of the “no change” CGI‐I group (*M* = 8.65), obtaining a ΔTHI‐MCID of 7.8. Thus, an MCID range of 7.8 to 12 was found for the THI. Considering the skewed distribution of the MCID results and remaining conservative, we suggest that the median of the range (ie, a reduction of 11 points on the THI) can be considered minimally clinically relevant.

For the TFI, a moderate effect size of Cohen's *d* = 0.64 [95% CI: 0.37, 0.91] was observed for the “minimally better” group after 12 weeks, while the “no change” group had a small effect size of Cohen's *d* = 0.26 [95% CI: 0.02, 0.51]. Cohen's *d*_anchor_ = 0.52 was multiplied by the pooled SD of the ΔTFI (18), which gives an effect size‐MCID of 9.4. Using the ROC method to determine the MCID, an AUC of 0.62 and an optimal cut‐off threshold (ROC‐MCID) of 5 points were identified. The mean change score of the “minimally better” group (13.2) and the mean change score of the “no change” group (5.9) resulted in a difference of 7.3 points (ΔTFI‐MCID). In summary, the anchor‐based estimates of the MCID ranged from 7.3 to 9.4 (ROC‐MCID is below SEM). Based on these results, we suggest that the more conservative of the 2 measures, namely a reduction of 9 points on the TFI, can be considered minimally clinically significant.

#### Influence of Time Interval

As a secondary analysis, MCID values were calculated after 6 and 36 weeks. The subsamples are described in Supplemental Tables S2 and S3, available online. Correlations with the CGI‐I were *ρ* = −0.27 (*P* < .001, ΔTHI) and *ρ* = −0.41 (*P* < .001, ΔTFI) after 6 weeks and *ρ* = −0.47 (*P* < .001, ΔTHI) and *ρ* = −0.52 (*P* < .001, ΔTFI) after 36 weeks. The distributions of ΔTHI/TFI according to CGI‐I are depicted in the Appendix (Supplemental Figures S1‐S4, available online). The MCID results are presented in Figures 3A and 4A. After 6 weeks, the MCID ranged from 7.8 to 12 for ΔTHI and is estimated at 7.4 for ΔTFI. After 36 weeks, the MCID ranged from 13 to 16 for ΔTHI and 10.1 to 12 for ΔTFI.

#### Influence of THI/TFI Baseline Severity

**Figure 5:**
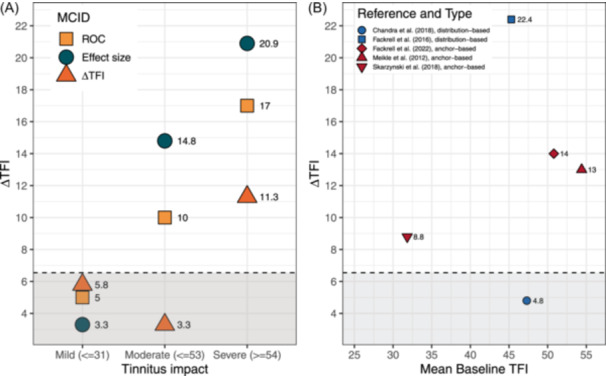
TFI‐MCID by tinnitus severity. (A) MCID results by tinnitus severity. (B) Literature MCID results by mean baseline TFI values (Table 1). The dashed line represents the standard error of measurement. MCID, minimal clinically important difference; TFI, Tinnitus Functional Index.

To investigate the influence of baseline THI/TFI severity on the MCID, both samples were divided into patients with low tinnitus impact, moderate tinnitus impact, and severe tinnitus impact at baseline. The (12 weeks) MCIDs were obtained with the anchor‐based approaches for all groups separately, and the results are depicted in Figures 3B and 5A. Correlations with the CGI‐I were *ρ* = −0.14 (*P* = .12, THI) and *ρ* = −0.32 (*P* = .004, TFI) for mild impact, *ρ* = −0.24 (*P* = .006, THI) and *ρ* = −0.32 (*P* = <.001, TFI) for moderate impact, and *ρ* = −0.45 (*P* < .001, THI) and *ρ* = −0.50 (*P* < .001, TFI) for severe impact. The distributions of ΔTHI/TFI according to CGI‐I are depicted in the Appendix (Supplemental Figures S5‐S10, available online). Among mild tinnitus impact, MCID ranged between 6 and 8.9 for THI. Among moderate impact, MCID ranged between 18 and 20.2 for THI and 10 and 14.8 for TFI. Among severe impacts, MCID ranged between 14.2 and 26.5 for THI and 11.3 and 20.9 for TFI.

## Discussion

The present study aimed to conduct a comprehensive psychometric analysis of the MCID for the THI and the TFI using different anchor‐based approaches. To achieve this, a representative dataset of 364 and 359 tinnitus patients (for THI and TFI, respectively) who underwent various standardized 12‐week treatments within a clinical trial conducted at 5 clinical sites across Europe was utilized.^26^

The main finding identifies an MCID range of 7.8 to 12 with a point estimate of 11 points difference in the THI as clinically relevant change after 12 weeks of treatment. For the TFI, an MCID range of 7.3 to 9.4 points with a point estimate of 9 points difference was analogously identified. Independent of the scale, the MCID is highly context‐specific and depends on baseline severity and time interval. Longer intervals and higher baseline severity require greater score reduction to be clinically meaningful.

Zeman et al's MCID of 7 points for the THI obtained with the effect size method^16^ is slightly lower than the range of 7.8 to 12 found in our study, with our effect size method yielding an MCID of 11 points. This difference likely stems from variations in ΔTHI distributions between studies, as Zeman's sample showed smaller ΔTHI values. Despite demographic similarities, differences in treatment type, duration, and combination therapies may explain the discrepancy,^4^, ^24^ with combination treatments in our sample showing higher ΔTHI values (Table S4). Additionally, Zeman's shorter treatment duration (4 weeks vs. 12 weeks) may contribute to the lower MCID, as we found a tendency for longer treatments to require larger score reductions to be clinically meaningful. Lastly, recent findings suggest a higher response rate for the THI (38.2%) compared to other tinnitus measures (TFI: 18.6%, Tinnitus Questionnaire [TQ]: 19.7%), supporting our finding of a higher THI‐MCID criterion than 7 points.^25^

The TFI‐MCID value proposed here is lower than Meikle et al's 13 points,^20^ as they used the “much improved” and “moderately improved” groups as anchors, while we used the “minimally better” group. We believe that choosing the “minimally better” group as the anchor group is conceptually more consistent with the MCID definition.^4^ Reanalyzing our data with the “much better” group yielded higher MCID estimates (12‐17.2 points, Figure S11), which supports our proposed MCID of 9 points and suggests the previous standard of 13 points as reflecting moderate rather than minimal improvement. Additionally, to allow for another direct comparison, we applied our method to Meikle et al's data resulting in a 9.1‐point estimate, which is much closer to our value. The MCID value of 8.8 points proposed by Skarżyński et al^23^ is close to our finding, but their estimate was based on ROC analysis comparing patients with “much” or “very much improvement” to the “no change” group. In addition, the mean change in the TFI score of the “minimally improved” group of 8.7 points was used as the MCID estimate. Applying our ΔTFI‐MCID method to their data resulted in a value of 9.57 points. Fackrell et al^24^ reported an MCID of 14 points based on a distribution‐based measure, but re‐evaluating their data using our ΔTFI‐MCID method resulted in a value of 8.8 points. Therefore, our MCID estimate of 9 points seems to be in line with the data from the literature when using a consistent method and the anchor “minimally improved” (ΔTFI‐MCID).

In addition, we observed that patients with higher baseline severity required larger reductions in both outcomes to perceive the change as clinically meaningful which is supported by previous findings.^24^, ^27^ This may partially be influenced by “regression to the mean” (ie, the statistical tendency for extreme values to become less extreme over time.^5^ Furthermore, it seems plausible that the clinical relevance of an absolute score change depends on the baseline value; a drop from 100 to 90 might appear far less meaningful than a drop from 10 to 0. Presenting changes in relative rather than absolute terms could mitigate this issue, as it inherently accounts for baseline differences (eg, 10% reduction vs. 100% reduction).

Similarly, we observed that with increasing time intervals, larger reductions in THI/TFI scores were required to be considered clinically meaningful. From a clinical perspective, it seems plausible that patients consider the time component when evaluating subjective clinical improvement. In other words, longer treatment intervals are expected to be more effective than shorter ones. However, this trend was much smaller than the baseline severity trend. Also, mixed results have been found previously for the TFI depending on the method used (ROC method: larger MCID with increasing time interval; ΔTFI method: larger MCID with decreasing time interval).^24^ We repeated the analysis after splitting the sample according to median tinnitus duration into a group with shorter tinnitus duration and a group with longer tinnitus duration to test for a potential influence of the natural history of tinnitus on the time dependence of the MCID, but no clear trend could be identified (Supplemental Tables S5 and S6, available online). These results highlight that MCID estimates can be strongly influenced by sample characteristics as well as study design. Other factors that may influence the MCID include tinnitus fluctuations and treatment type.^4^, ^22^, ^23^ Additionally, the heterogeneity in biological underpinnings of tinnitus might further complicate the generation of consistent MCID estimates.

These findings highlight the challenge of establishing a universally valid MCID. From a methodological perspective, it has been argued that the use of a single MCID value may be misguided due to the wide range of calculation methods and the dependence on the sample.^7^ On the other hand, a certain agreement by the research community about MCID values for questionnaires is needed for the planning and analysis of clinical trials and the establishment of approval criteria for specific interventions. Therefore, it is recommended that the MCID value should be triangulated based on the results of different estimation methods, supported by clinical trial experience, to arrive at a narrow range or single MCID value that needs to be confirmed by multiple studies.^40^, ^41^ Even so, researchers should avoid simply “plugging in” an MCID value without consideration of context. The variability across patient populations and clinical contexts needs to be acknowledged and consensus recommendations on the most adequate anchor‐based methods are needed to improve comparability and achieve generally accepted MCID estimates in the tinnitus field.

In terms of limitations, it should be noted that the group reporting “no change” on the CGI showed an average improvement on both THI and TFI. Moreover, the correlation between CGI‐I and ΔTHI/TFI was only small to moderate in our sample, which may limit the use of CGI‐I as an appropriate anchor. Similarly, the AUC of the ROC analysis was below the proposed threshold of 0.7.^6^ On the other hand, our study has particular strengths over previous studies. First, we investigated a large sample from a multicentric study involving the most widely used therapeutic interventions for tinnitus. Second, we determined MCIDs for the 2 most widely used TQs in the same sample and for various time points. Further investigation in other study cohorts, ideally using the same anchor‐based approaches, is needed to validate our results.

## Conclusion

Based on our results, we suggest that a 11‐point reduction in the THI and a 9‐point reduction in the TFI is clinically meaningful. When selecting an appropriate MCID value for a given context, factors such as sample size calculation, baseline severity, and time interval are important factors to consider. To improve the comparability of results, a consensus on the optimal anchor‐based MCID approaches is required.

## Author Contributions

**Milena Engelke**, conceptualization, methodology, formal analysis, visualization, writing–original draft; **Laura Basso**, conceptualization, methodology, formal analysis, visualization, writing–original draft; **Berthold Langguth**, supervision, conceptualization, writing–review and editing; **Florian Zeman**, methodology, writing–review and editing; **Winfried Schlee**, project administration, funding acquisition, writing–review and editing; **Stefan Schoisswohl**, project administration, writing–review and editing; **Rilana Cima**, writing–review and editing; **Dimitris Kikidis**, funding acquisition, writing–review and editing; **Jose Antonio Lopez‐Escamez**, writing–review and editing; **Petra Brüggemann**, writing–review and editing; **Birgit Mazurek**, writing–review and editing; **Jorge Piano Simões**, supervision, conceptualization, methodology, writing–review and editing.

## Disclosures

### Competing interests

None.

### Funding source

This project has received funding from the European Union's Horizon 2020 Research and Innovation Programme, Grant Agreement Number 848261. The funders had no role in study design, data collection and analysis, decision to publish, or preparation of the manuscript.

## Supporting information

Supporting information.
