# Supplementary material for: Estimation of Minimal Clinically Important Difference for Tinnitus Handicap Inventory and Tinnitus Functional Index
Source: Otolaryngol Head Neck Surg. 2025 Mar 20;173(1):69–79. doi: 10.1002/ohn.1217 (PMC12207346; doi:10.1002/ohn.1217)
Supplement: Supplementary file 1 — Supporting information. [file OHN-173-69-s001.pdf]

Supplementary Information

Names of the clinical sites

(1) University of Regensburg, Regensburg, Germany (RCT coordinator)

(2) Charité – Universitaetsmedizin Berlin, Berlin, Germany

(3) Ethniko Kai Kapodistriako Panepistimo Athinon, Athens, Greece

(4) Hospital Universitario Virgen de las Nieves/Hospital Clinico Universitario San Cecilio, Granada, Spain

(5) Katholieke Universiteit Leuven, Leuven, Belgium

Study protocol[1]

Please find detailed information about the design of the trial in the study protocol[1].

Test-retest reliability of the TFI

As the intraclass correlation coefficient (ICC) should be used for test-retest reliability in the above SEM formula[2], only ICC values were considered. Specifically, the ICC values of reproducibility studies by [3], [4], [5], [6], [7], [8] and [9] were weighted by the respective sample sizes and averaged. This resulted in r=0.89, which was used to calculate the SEM.

Table S1. Test-retest reliability values of the TFI in the literature

Country	Value	N	Method	Reference
**US**	0.78	37	Not specified	Meikle et al. (2012) [10]
**UK – general public**	0.91	44	ICC	Fackrell et al. (2016) [3]
**Sweden**	0.88	259	ICC	Müller et al. (2016) [4]
**Sweden**	0.93	54	ICC	Hoff & Kähäri (2017) [5]
**Switzerland**	0.91	86	Pearson’s correlation	Peter et al. (2017) [11]
**New Zealand**	0.91	40	ICC	Chandra et al. (2018) [7]
**UK – clinical population**	0.87	50	ICC	Fackrell et al. (2018) [6]
**China**	0.84	25	ICC	Kam et al. (2018) [8]
**Japan**	0.86	41	ICC	Suzuki et al. (2019) [9]
**Italy**	0.83	57	Alpha coefficient	Barozzi et al. (2020) [12]
**Denmark**	0.848	23	Pearson’s correlation	Hald et al. (2022) [13]
**Spain**	1 (CI: 0.99-1)	18	ICC(2,1)	Soriano-Reixach et al. (2023) [14]

Note. Studies found in the literature reporting test-retest reliability values for TFI. Only ICC values were considered to obtain an averaged estimate (weighted by sample size) to be used for the calculation of the SEM. Only [14] specify the type of ICC used.

Table S2. Baseline characteristics of the subsample for the 6-week change analysis

	THI sample (N = 338)	TFI sample (N = 332)
**Characteristic**	**N (%) / Mean ± SD**	
Sex – no. (%)		
Female	146 (43.2%)	143 (43.1%)
Male	192 (56.8%)	189 (56.9%)
Age (years)	51.7 ± 12.4	51.7 ± 12.3
Tinnitus duration (months)*	96 ± 156*	122 ± 112*
THI/TFI score	48.5 ± 19.9	49.6 ± 20.6
PHQ-9 score	7.3 ± 4.9	7.3 ± 4.9

Note. Plus–minus values are means ± SD; *only for Tinnitus duration median ± IQR is used due to non-normally distributed data. THI = Tinnitus Handicap Inventory; TFI = Tinnitus Functional Index; PHQ-9 = Patient Health Questionnaire for Depression.

Table S3. Baseline characteristics of the subsample for the 36-week change analysis

	THI sample (N = 300)	TFI sample (N = 295)
**Characteristic**	**N (%) / Mean ± SD**	
Sex – no. (%)		
Female	133 (44.3%)	131 (44.4%)
Male	167 (55.7%)	164 (55.6%)
Age (years)	51.8 ± 12.6	51.7 ± 12.6
Tinnitus duration (months)*	108 ± 166.5	126 ± 113
THI/TFI score	47.3 ± 19.6	48.6 ± 20.5
PHQ-9 score	7.2 ± 4.9	7.21 ± 4.9

Note. Plus–minus values are means ± SD; *only for Tinnitus duration median ± IQR is used due to non-normally distributed data. THI = Tinnitus Handicap Inventory; TFI = Tinnitus Functional Index; PHQ-9 = Patient Health Questionnaire for Depression.

Figure S1. ΔTHI distribution at CGI-I after 6 weeks

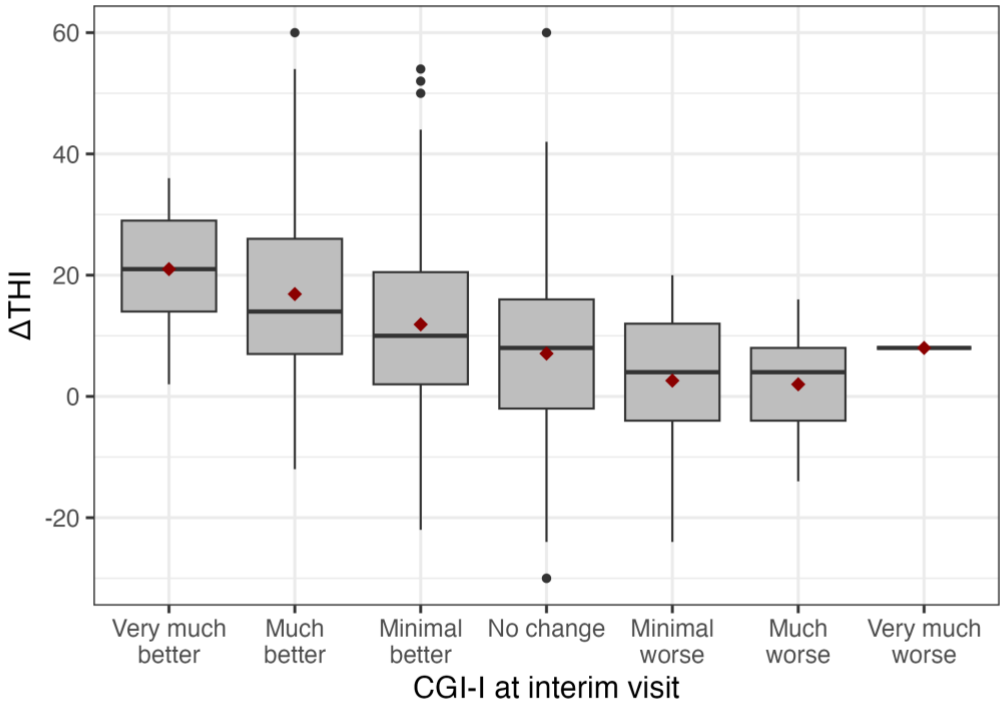

Note. Error bars indicate whiskers (standard boxplot), and red squares show the group mean. ΔTHI = baseline THI – THI after 6 weeks. CGI-I = Clinical Global Impression Scale – Improvement; THI = Tinnitus Handicap Inventory.

Figure S2. ΔTHI distribution at CGI-I after 36 weeks

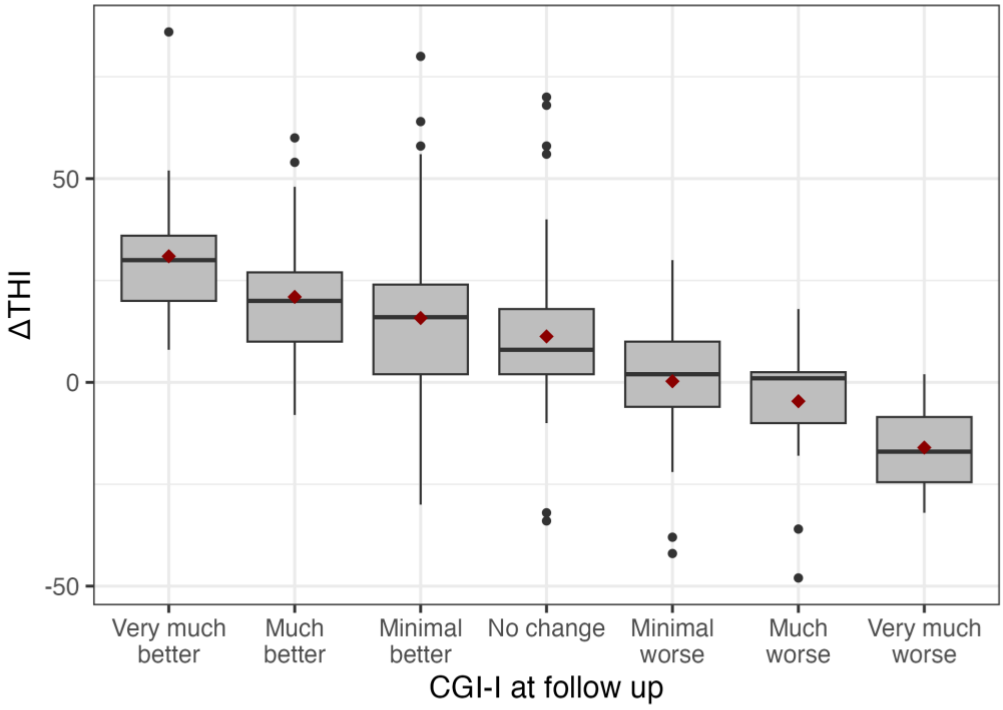

Note. Error bars indicate whiskers (standard boxplot), and red squares show the group mean. ΔTHI = baseline THI – THI after 36 weeks. CGI-I = Clinical Global Impression Scale – Improvement; THI = Tinnitus Handicap Inventory.

Figure S3. ΔTFI distribution at CGI-I after 6 weeks

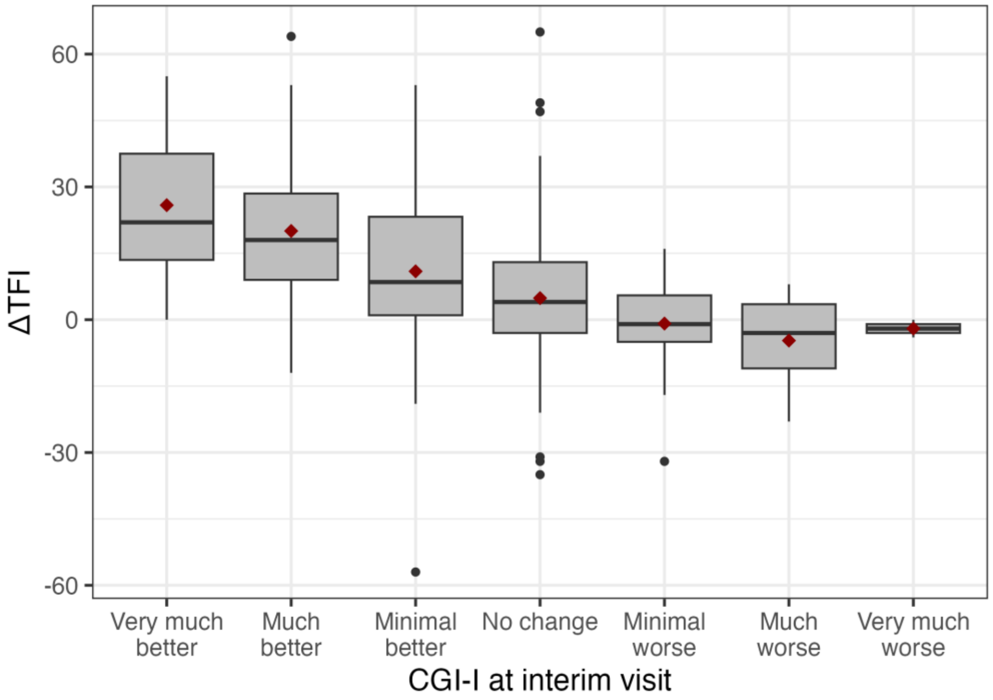

Note. Error bars indicate whiskers (standard boxplot), and red squares show the group mean. ΔTFI = baseline TFI – TFI after 6 weeks. CGI-I = Clinical Global Impression Scale – Improvement; TFI = Tinnitus Functional Index.

Figure S4. ΔTFI distribution at CGI-I after 36 weeks

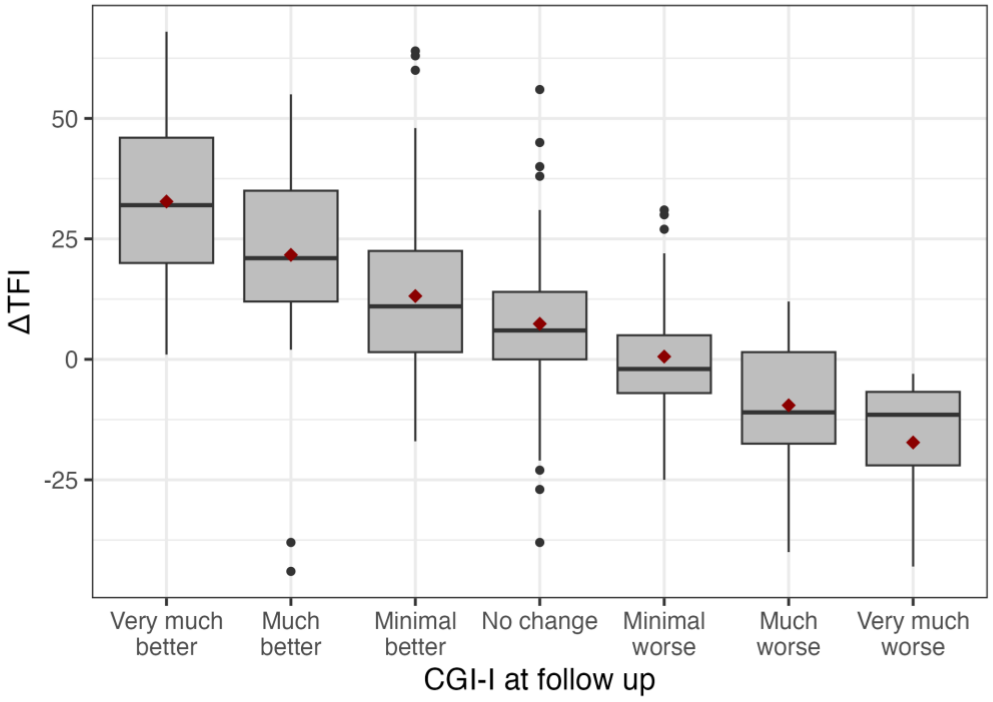

Note. Error bars indicate whiskers (standard boxplot), and red squares show the group mean. ΔTFI = baseline TFI – TFI after 36 weeks. CGI-I = Clinical Global Impression Scale – Improvement; TFI = Tinnitus Functional Index.

Figure S5. ΔTHI distribution at CGI-I after 12 weeks (mild THI baseline)

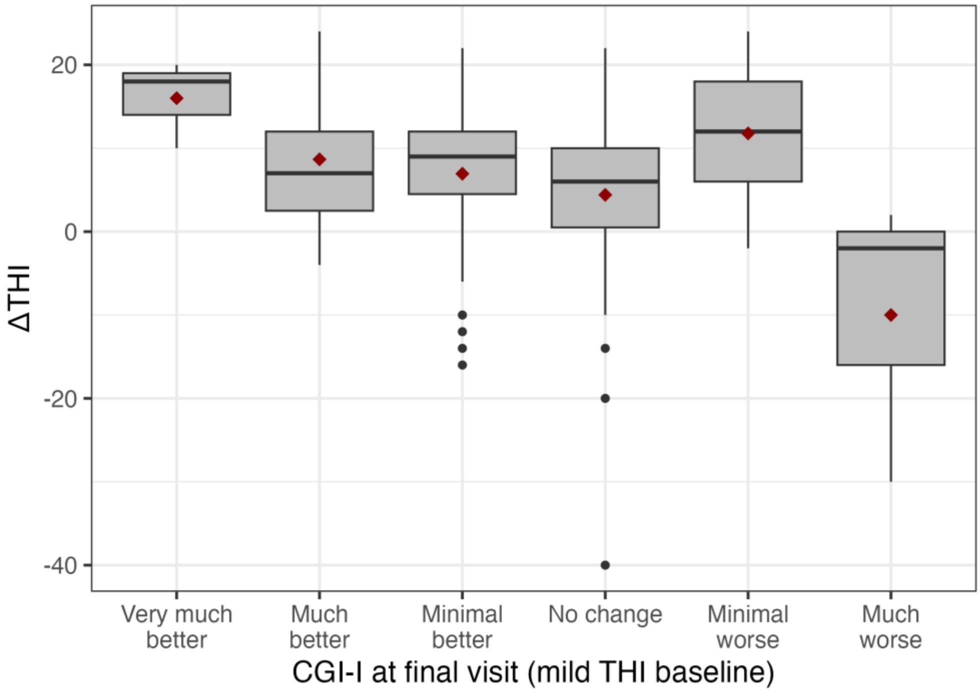

Note. Patients with mild tinnitus impact at baseline (THI ≤ 36). Error bars indicate whiskers (standard boxplot), and red squares show the group mean. ΔTHI = baseline THI – THI after 12 weeks.
CGI-I = Clinical Global Impression Scale – Improvement; THI = Tinnitus Handicap Inventory.

Figure S6. ΔTHI distribution at CGI-I after 12 weeks (moderate THI baseline)

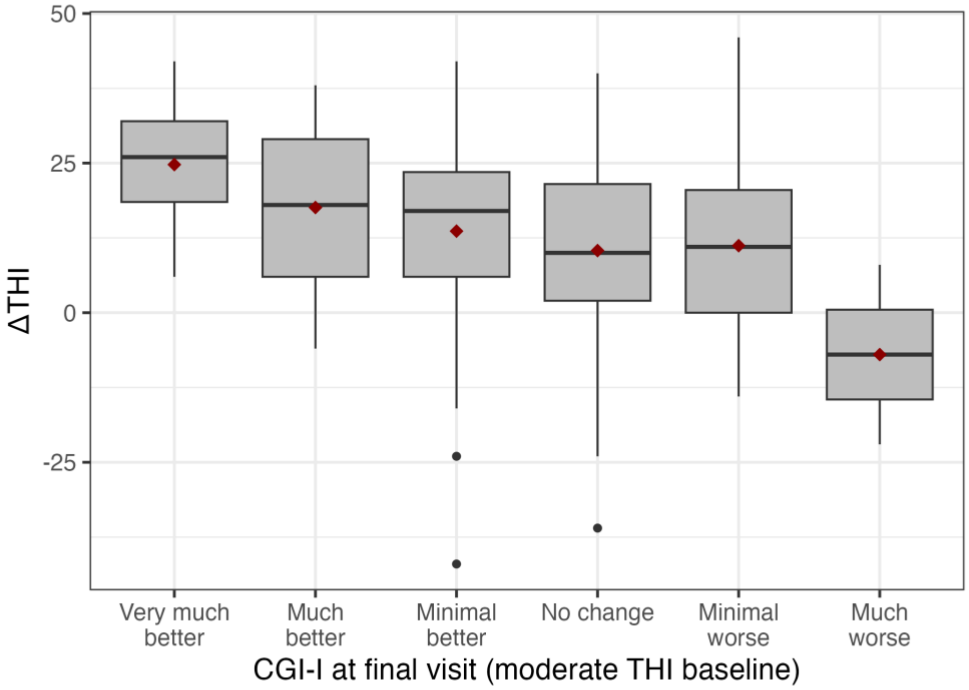

Note. Patients with moderate tinnitus impact at baseline (THI ≤ 56). Error bars indicate whiskers (standard boxplot), and red squares show the group mean. ΔTHI = baseline THI – THI after 12 weeks.
CGI-I = Clinical Global Impression Scale – Improvement; THI = Tinnitus Handicap Inventory.

Figure S7. ΔTHI distribution at CGI-I after 12 weeks (severe THI baseline)

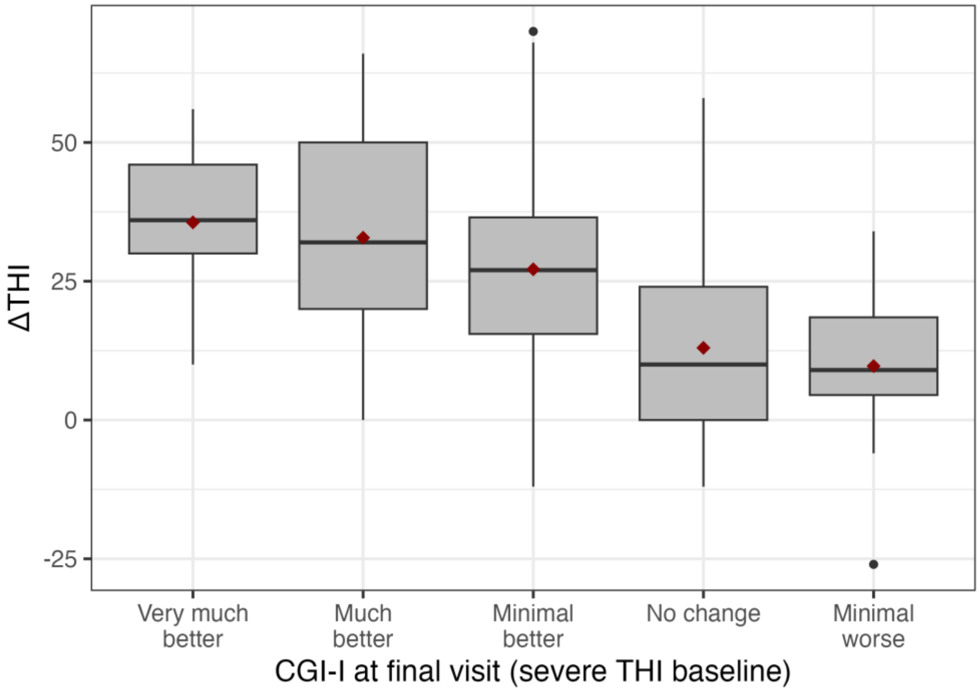

Note. Patients with severe tinnitus impact at baseline (THI ≥ 58). Error bars indicate whiskers (standard boxplot), and red squares show the group mean. ΔTHI = baseline THI – THI after 12 weeks.
CGI-I = Clinical Global Impression Scale – Improvement; THI = Tinnitus Handicap Inventory.

Figure S8. ΔTFI distribution at CGI-I after 12 weeks (mild TFI baseline)

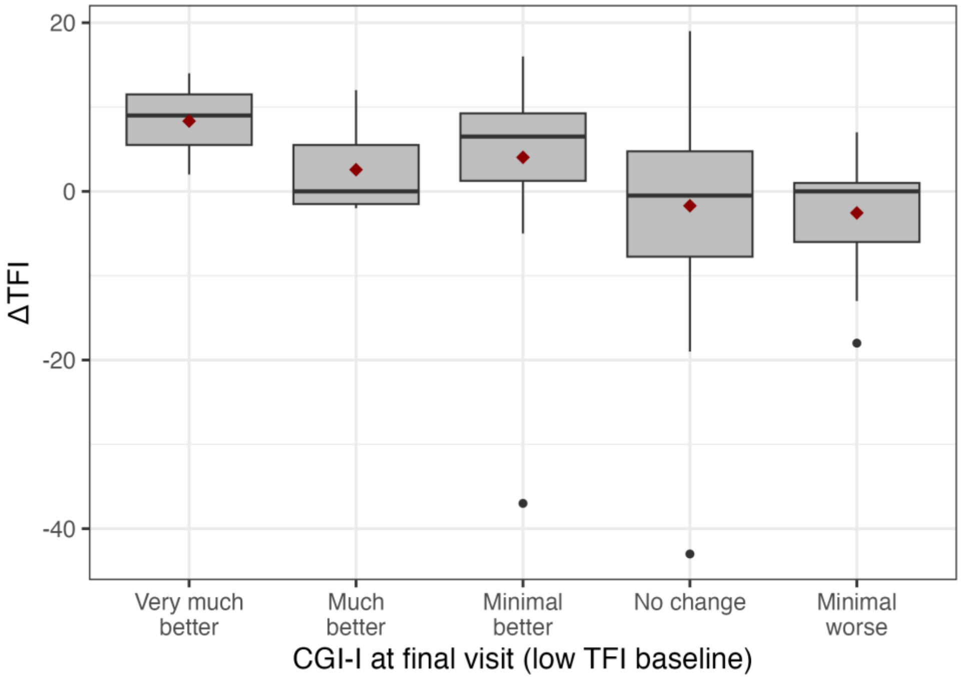

Note. Patients with mild tinnitus impact at baseline (TFI ≤ 31). Error bars indicate whiskers (standard boxplot), and red squares show the group mean. ΔTFI = baseline TFI – TFI after 12 weeks.
CGI-I = Clinical Global Impression Scale – Improvement; TFI = Tinnitus Functional Index.

Figure S9. ΔTFI distribution at CGI-I after 12 weeks (moderate TFI baseline)

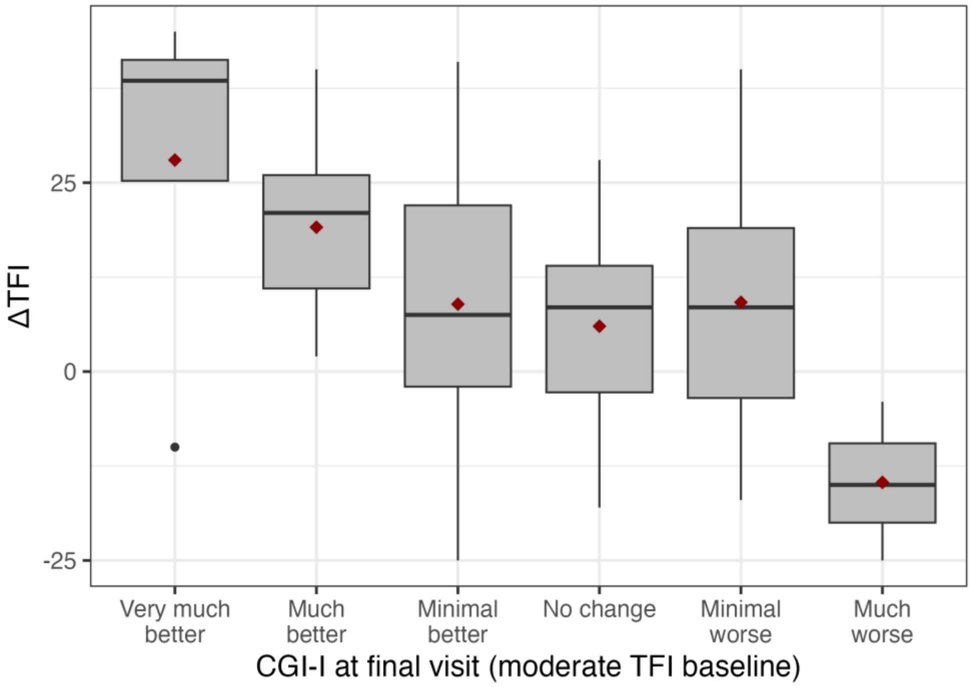

Note. Patients with moderate tinnitus impact at baseline (TFI ≤ 53). Error bars indicate whiskers (standard boxplot), and red squares show the group mean. ΔTFI = baseline TFI – TFI after 12 weeks.
CGI-I = Clinical Global Impression Scale – Improvement; TFI = Tinnitus Functional Index.

Figure S10. ΔTFI distribution at CGI-I after 12 weeks (severe TFI baseline)

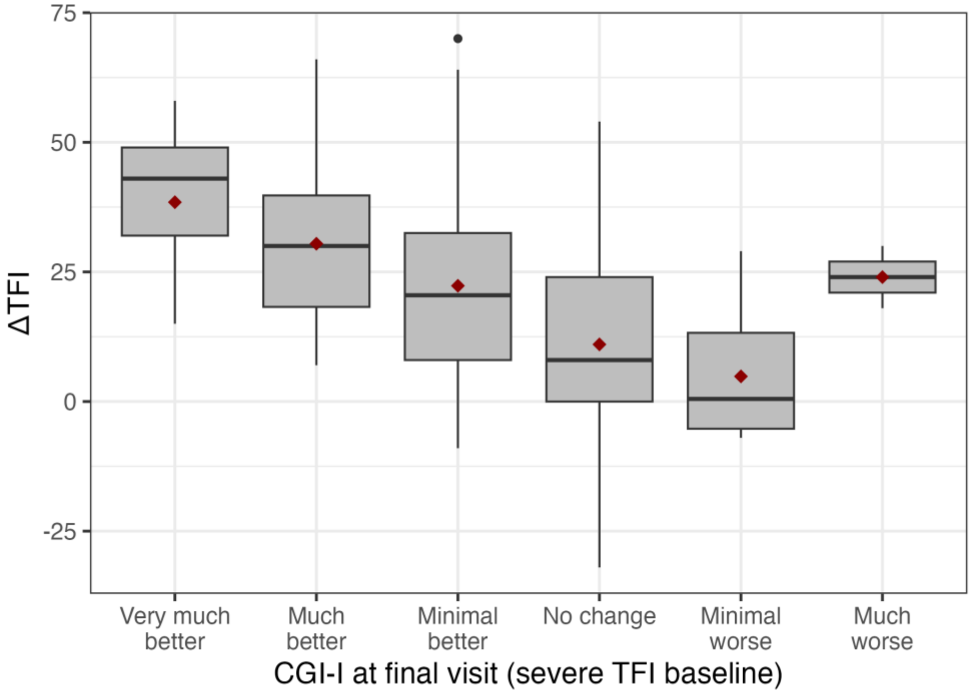

Note. Patients with severe tinnitus impact at baseline (TFI ≥ 54). Error bars indicate whiskers (standard boxplot), and red squares show the group mean. ΔTFI = baseline TFI – TFI after 12 weeks.
CGI-I = Clinical Global Impression Scale – Improvement; TFI = Tinnitus Functional Index.

Table S4. ΔTHI for single and combination treatments

Single treatments	6.0 ± 15.1
Combination treatments	11.6 ± 13.3

Note. Plus–minus values are means ± SD; THI = Tinnitus Handicap Inventory; ΔTHI = baseline THI – THI after 12 weeks.

Figure S11. MCID results based on the "much better" group (CGI-I = 2) as anchor group (A) and literature findings (B)

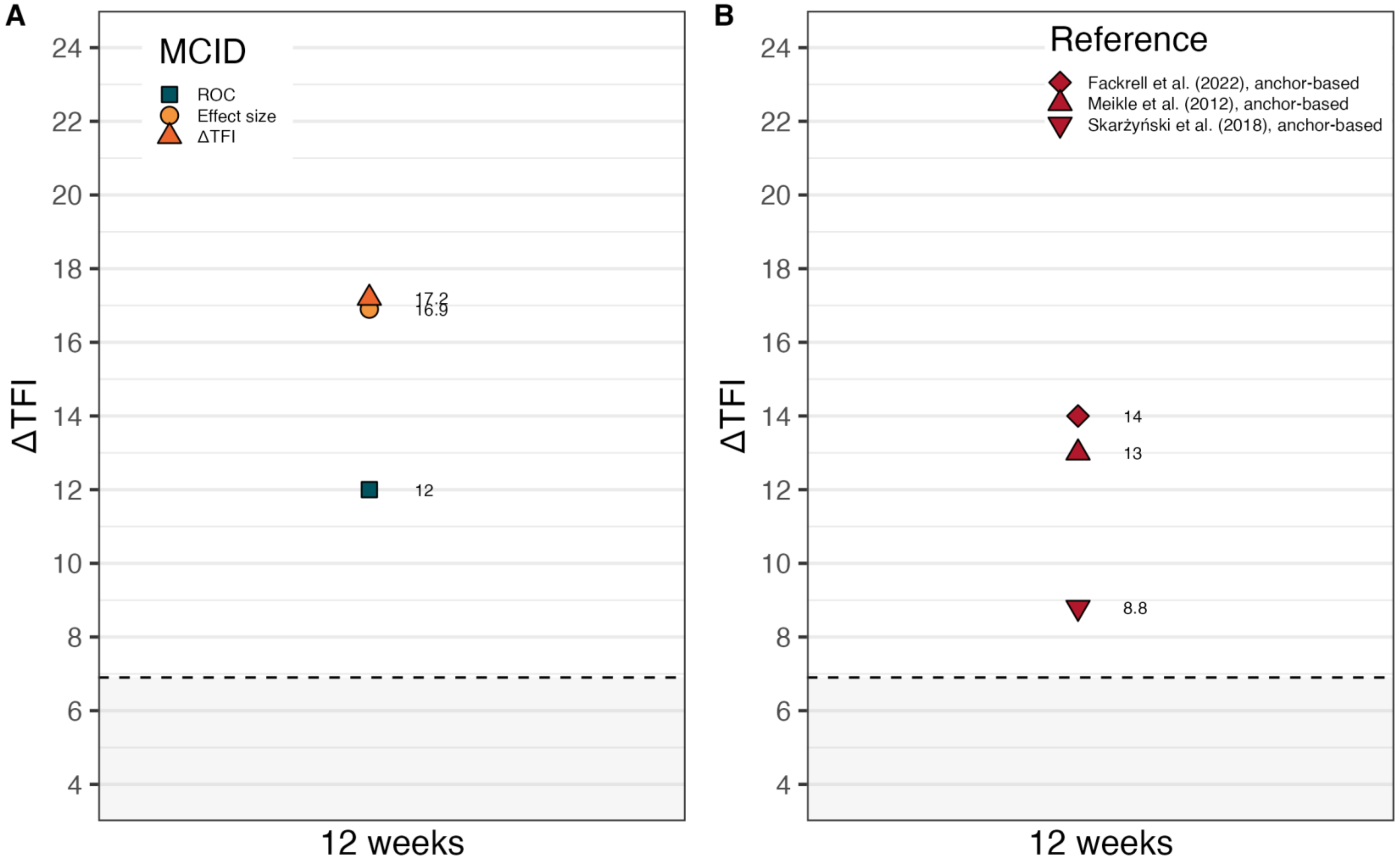

Table S5. THI-MCID estimates at 36 weeks according to tinnitus duration

	Shorter tinnitus duration (< 96 months)	Longer tinnitus duration (≥ 96 months)
ΔTHI	3.6	3.1
Effect size	20.3	12.8
ROC	16	16

Note. The sample was split according to a median split of tinnitus duration (96 months).

Table S6. TFI-MCID estimates at 36 weeks according to tinnitus duration

	Shorter tinnitus duration (< 96 months)	Longer tinnitus duration (≥ 96 months)
ΔTFI	6.2	4.3
Effect size	14.5	12
ROC	11	14

Note. The sample was split according to a median split of tinnitus duration (96 months).

References

1. Schoisswohl S, Langguth B, Schecklmann M, et al. Unification of Treatments and Interventions for Tinnitus Patients (UNITI): a study protocol for a multi-center randomized clinical trial. *Trials*. 2021;22(1):875. doi:10.1186/s13063-021-05835-z
2. Vet HCW de, Terwee CB, Mokkink LB, Knol DL. *Measurement in Medicine: A Practical Guide*. Cambridge University Press; 2011.
3. Fackrell K, Hall DA, Barry JG, Hoare DJ. Psychometric properties of the Tinnitus Functional Index (TFI): Assessment in a UK research volunteer population. *Hearing Research*. 2016;335:220-235. doi:10.1016/j.heares.2015.09.009
4. Müller K, Edvall NK, Idrizbegovic E, et al. Validation of Online Versions of Tinnitus Questionnaires Translated into Swedish. *Frontiers in Aging Neuroscience*. 2016;8. Accessed February 13, 2024. https://www.frontiersin.org/articles/10.3389/fnagi.2016.00272
5. Hoff M, Kähäri K. A Swedish cross-cultural adaptation and validation of the Tinnitus Functional Index. *International Journal of Audiology*. 2017;56(4):277-285. doi:10.1080/14992027.2016.1265154
6. Fackrell K, Hall DA, Barry JG, Hoare DJ. Performance of the Tinnitus Functional Index as a diagnostic instrument in a UK clinical population. *Hearing Research*. 2018;358:74-85. doi:10.1016/j.heares.2017.10.016
7. Chandra N, Chang K, Lee A, Shekhawat GS, Searchfield GD. Psychometric Validity, Reliability, and Responsiveness of the Tinnitus Functional Index. *J Am Acad Audiol*. 2018;29(07):609-625. doi:10.3766/jaaa.16171
8. Kam ACS, Leung EKS, Chan PYB, Tong MCF. Cross-cultural adaptation and psychometric properties of the Chinese tinnitus functional index. *International Journal of Audiology*. 2018;57(2):91-97. doi:10.1080/14992027.2017.1375162
9. Suzuki N, Oishi N, Ogawa K. Validation of the Japanese version of the tinnitus functional index (TFI). *International Journal of Audiology*. 2019;58(3):167-173. doi:10.1080/14992027.2018.1534279
10. Meikle MB, Henry JA, Griest SE, et al. The Tinnitus Functional Index: Development of a New Clinical Measure for Chronic, Intrusive Tinnitus. *Ear and Hearing*. 2012;33(2):153. doi:10.1097/AUD.0b013e31822f67c0
11. Peter N, Kleinjung T, Jeker R, Meyer M, Klaghofer R, Weidt S. Tinnitus functional index: validation of the German version for Switzerland. *Health Qual Life Outcomes*. 2017;15(1):94. doi:10.1186/s12955-017-0669-x
12. Barozzi S, Del Bo L, Passoni S, et al. Psychometric properties of the Italian Tinnitus Functional Index (TFI). *Acta Otorhinolaryngol Ital*. 2020;40(3):230-237. doi:10.14639/0392-100X-2432
13. Hald MO, Dreyer R, Hald M, Mikkelsen S, Ovesen T. Validation of the Danish version ofthe Tinnitus Functional Index. *Dan Med J*. 2022;69(10):A02220135.
14. Soriano-Reixach MM, Sampedro JJN, Minguez MSG, Rey-Martínez J, Altuna X. Translation into Spanish and validation of the Tinnitus Functional Index (TFI). *Acta Otorrinolaringologica (English Edition)*. 2023;74(5):305-314. doi:10.1016/j.otoeng.2023.03.003
